# Development of a CanMEDS-based instrument for evaluating medical students’ perceptions of the key competencies of a socially accountable healthcare practitioner

**DOI:** 10.1007/s40037-020-00564-6

**Published:** 2020-02-07

**Authors:** Claudia Naidu, Steve Reid, Vanessa Burch

**Affiliations:** 1 Primary Health Care Directorate, Faculty of Health Sciences, University of Cape Town, Cape Town, South Africa; 2 Department of Medicine, Faculty of Health Sciences, University of Cape Town, Cape Town, South Africa

**Keywords:** CanMEDS, Factor analysis, Medical students, Social accountability, Scale development, South Africa

## Abstract

**Introduction:**

Numerous frameworks and tools have been developed to assist medical schools striving to achieve their social accountability mandate. The purpose of this study was to design an instrument to evaluate medical students’ perceptions of the key competencies of a ‘socially accountable’ healthcare practitioner using widely accepted frameworks which contain clear measurable outcomes.

**Methods:**

The instrument was designed in three phases: selection of a competency-based framework, development of items, and validation of the instrument through exploratory factor analysis. Medical students in the 6‑year medical degree program at the University of Cape Town, South Africa were invited to participate in the study. Descriptive and inferential statistical analysis was performed using Stata/SE version 13.1.

**Results:**

Of 619 students invited to participate in the study, 484 (78%) responded. The CanMEDS framework was selected for designing the instrument, which comprised 35 statements reflecting five competencies for each CanMEDS role. Exploratory factor analysis of the student responses yielded a 28-item instrument. There was a significant difference in overall Perceptions of Social Accountability Instrument (PSAI) scores between men and women (*p* = 0.002) but no significant difference between the overall PSAI scores for students in the respective years of study.

**Discussion:**

This study describes the design of an instrument to evaluate medical students’ perceptions of the essential competencies of socially accountable healthcare practitioners. Used longitudinally, the data may provide evidence of the successes of our programs and identify areas where further improvements are required.

**Electronic supplementary material:**

The online version of this article (10.1007/s40037-020-00564-6) contains supplementary material, which is available to authorized users.

## Introduction

Globally there is an ongoing call for medical education to focus on the interests of patients and populations [1]. Numerous frameworks, guides and tools have been developed to assist medical schools to achieve this mandate [2–8]. These initiatives aim to measure institutional progress towards achieving the social accountability agenda [9], being ‘the obligation [of medical schools] to direct their education, research and service activities towards addressing the priority health concerns of the community, region, and/or nation they have a mandate to serve, as jointly identified by governments, healthcare organizations, health professionals and the public’[2].

The literature distinguishes between two closely related topics, the ‘big picture’—social accountability of institutions and an ‘on the ground reality’—the socially accountable behaviour and practices of individual healthcare graduates [9]. Our understanding of the latter is, however, limited [10, 11] and indicators to measure the extent to which this is achieved are largely limited to the choice of practice location and speciality of graduates.

Guided by the fundamental principles representative of a socially accountable model of healthcare, the values of relevance, quality, equity and partnerships [12] are expected to underpin the behaviour and practices of a socially accountable healthcare professional. The envisioned product is a doctor responsive to the priority health needs of society, who conducts him/herself in a socially responsible manner from both a personal and professional perspective, and holds him/herself accountable to society for providing healthcare which is comprehensive, relevant, accessible, patient-centred, responsive and cost-effective [13]. This entails reflection, engagement, advocacy, leadership, and the ability and preparedness to serve.

Generally students prioritise the three C’s of medical care desired by patients—Caring (compassion, empathy, patient-centred), Communication, including listening, and Competence (knowledge and ‘being a professional’) [14]. However, some of these positive attitudes decline over time [15, 16]. Furthermore, the idea of a social contract, an obligation to act altruistically, care for indigent patients, reduce health inequalities, and address priority health concerns is not uniformly endorsed by clinical trainees [17, 18], highlighting the critical need to better understand students’ perceptions of the attributes of a socially accountable healthcare practitioner and how these may change and evolve over time, both before and after graduation. While we are starting to develop an understanding of what works, there is no robust way to track the process to ensure that we arrive at the desired outcome—graduates who are adequately prepared with the necessary skills and values to respond to the diverse and ever-changing health needs of the population [19].

An early (designed in the 1970s) largely unused tool, the Attitudes Toward Social Issues in Medicine (ATSIM) scale [20], measured non-academic factors thought to predispose students to good medical practice and this was followed by the Medical Students Attitudes towards the Underserved (MSATU) scale which was developed two decades later, aiming to measure students’ attitudes towards society’s responsibility to provide healthcare, physicians’ involvement in community health efforts, and healthcare available to indigent patients [21]. Since then the dehumanisation of healthcare [20] has sparked interest in developing tools focusing on professionalism and empathy rather than social accountability. All of these tools hint at the possibility of tracking students’ perceptions during training to gain an earlier understanding of the impact of the educational process they are engaged in and how this may ultimately influence their behaviour both during, and after, completing undergraduate training. To date, this has only been done to a limited extent and none of the work has specifically focused on a more formal construct of the socially accountable healthcare practitioner, as defined in existing competency frameworks [22]. Using outcomes frameworks to develop longitudinal measures of medical trainees’ perceptions of competencies during rather than at the end of training, would enable a proactive rather than reactive approach to curriculum design.

## Context

This study took place at the University of Cape Town (UCT), between 2012 and 2013. The UCT MB ChB is a 6-year training program, the first few years of which are primarily theory-based with limited clinical exposure and the remaining 2–3 years based in clinics, hospitals and other health facilities in the region. The training years are categorised into semesters, with the first 4 semesters (years 1 and 2) primarily based at medical school and focused on the teaching of basic sciences, and basic medical sciences. This is followed by rotations through disciplines during semesters 5 to 8 (years 3 and 4), designed to expand and consolidate the clinical skills developed in the earlier semesters of the curriculum with the final 2 years (semesters 9 to 12) largely spent as student interns at hospitals, rotating through clinical departments.

The purpose of this study was to design an instrument to longitudinally evaluate medical students’ perceptions of the key competencies of a ‘socially accountable’ healthcare practitioner, over the duration of their studies. Historically this definition is expressed as competencies in existing frameworks, and largely reflects the opinion of healthcare practitioners and educators. Exploring how strongly (or not) medical students identify with these competencies facilitates an evidence-driven (measured) approach to exploring students’ perceptions to guide curriculum design aimed at addressing the social accountability agenda.

## Methods

The instrument was designed in three phases: selection of a competency-based framework for developing an instrument which articulates the key competencies of a socially accountable doctor, development of items based on the selected framework, and exploration of medical students’ perceptions of these competencies using factor analysis.

### Selection of a competency-based framework

The CanMEDS framework was selected because it was developed to address calls for greater social responsiveness in medicine[23], has been widely adopted for program accreditation including in South Africa [24], and has well-defined, observable competencies that are suitable for developing a quantitative instrument. Furthermore, CanMEDS has been used to develop several questionnaires evaluating medical trainees’ opinions regarding the importance of the competencies contained in the framework [25].

### Instrument development

The first author selected 55 key/enabling competencies from the CanMEDS framework for potential inclusion in the instrument. Items were selected if they described competencies consistent with prevailing descriptions of the socially accountable healthcare practitioner [26, 27]. Through an iterative process of selection, checking, discussion, reselection, and final agreement on items, all authors used the selected competencies to formulate 35 statements reflecting the five competencies for each CanMEDS role, i.e. dimension that explicitly describes socially responsive, responsible and accountable behaviours/attributes. The dimensions were envisioned to be true domains of the construct of social accountability in order to allow for an aggregate model where a multidimensional construct is formed as an aggregate of its dimensions [28].

Cognitive interviews were held with a sample of 15 fourth-year medical students (2011 cohort) as a focus group to ensure readability and comprehension [29]. Each item had a 5-point Likert response scale ranging from: not important (1), slightly important (2), important (3), very important (4) and essential (5). Negatively worded items were reverse coded. The instrument was limited to 35 items because long surveys are associated with poor completion rates [30] and many scales/inventories have been significantly shortened in other studies without compromising quality and purpose, such as the Clinical Research Appraisal Inventory [31] and the Caring Behaviours Inventory [32].

### Validity

Validity is explained in terms of ‘whether the research truly measures that which it is intended to measure or how truthful the results are’ [33]. Construct validity is considered to be ‘the whole of validity’ as per the Standards of Educational and Psychological Measurement, with multiple facets [34]. With regards to establishing validity evidence in this study, the internal structure of the instrument (relating to the statistical or psychometric characteristics) and its relationship to other variables (i.e. other existing well-established validated measures or a ‘gold standard’ [35]) was determined. The former was performed through a factor analysis, which is one way to determine internal structure, and provide evidence of validity [36], the latter by comparing the scores with those on the MSATU scale introduced earlier [37]. With permission from the authors, 10 items from the MSATU scale [15] were included in the questionnaire administered to participants. The selected items described the behaviour or attitudes of a socially accountable doctor and were rated using a 5-point Likert response scale ranging from completely disagree (1) to completely agree (5).

### Instrument exploration

#### Participants

Medical students in the 6‑year school-leavers program at the University of Cape Town, South Africa were invited to participate in the study. Fourth-year students were invited to join the pilot study in 2012. All first, third and final-year students were invited to participate in the main study in 2013.

#### Data collection

In 2012 the 35-item instrument was piloted with the class of fourth-year medical students to test the user-friendliness of the instrument. This class was chosen due to easy access to the students on the first day of the new academic year. In 2013 the 35-item instrument and the 10 items from the MSATU scale were incorporated into a hardcopy survey administered to first-, third- and sixth-year medical students. The survey consisted of six sections: personal/demographic information (6 items); perspectives on competencies of healthcare professionals (45 items); career plans (7 items), education experiences (4 items), background information on family and schooling (5 items), and free text comment boxes (3 items) asking respondents to list five attributes they consider most important to being a ‘good’ doctor in their perspective and to expand on any of their views provided during completion of the questionnaire. The phrase ‘good’ doctor was used as initial focus groups and literature have demonstrated students’ lack of understanding of the term ‘social accountability’ and the authors wanted to avoid influencing, directing or priming respondents on their responses.

#### Study procedure

At the beginning of the academic year, first-year students were recruited to the study during a compulsory whole-class computer lab evaluation session and third- and sixth-year students, in groups of 30–40, were approached during their orientation sessions or class lectures, in their respective academic blocks. CN and in a few instances VB, who third- and sixth-year students would have been familiar with as educators in the faculty, administered the survey, starting with an explanation on the purpose of the study. Students were given an opportunity to ask any questions after which a hardcopy of the survey was distributed and completed following signed consent. Students were informed that their responses would be anonymised. Completed surveys were collected at the end of the sessions before students left the venue. Participants did not receive any compensation. The study was approved by the Human Research Ethics Committee of the University of Cape Town (HREC 199/2012). All procedures performed in studies involving human participants were in accordance with the ethical standards of the institutional and/or national research committee and with the 1964 Helsinki Declaration and its later amendments or comparable ethical standards.

### Data analysis

All pilot and survey data were entered into an Access database and exported as Excel spreadsheets for analysis using SPSS version 21 (IBM Corp., USA). Exploratory factor analysis (EFA) was performed using the data from the 35-item instrument in 2012 (pilot study) and the 45-item survey in 2013 (main study). Descriptive and inferential statistical analysis was only performed on data from the main study that included the 35-item instrument and the 10 items from the MSATU.

A maximum-likelihood EFA was performed, and a Promax rotation selected as it was expected that the factors would correlate as is consistent with social science research [38]. The Kaiser-Meyer-Olkin measure of sampling adequacy (MSA) and the Bartlett test of sphericity were calculated to ensure suitability of the data for EFA. The eigenvalue-greater-than-one test and the scree plot were used to identify the number of resulting factors. Items loading >0.3 were retained and grouped according to where they loaded highest on an identified factor. High cross-loading items and/or items that did not load on any factor at the 0.3 threshold were excluded. Cronbach alpha was calculated for each subscale and the total instrument along with the component correlations between all factor pairs.

As discussed in the literature, ‘multidimensional constructs can be conceptualised under an overall abstraction’ as long as the constructs have been well-defined, i.e. the relationship between the concept and the constructs has been specified [28, 39]. In this study, the subscales or dimensions of social accountability, as the aggregate construct, are themselves constructs conceived as specific components of the general construct they collectively constitute [28, 40], therefore scores can be analysed and reported on the dimension and overall construct level. The Perceptions of Social Accountability Instrument (PSAI) overall scores were calculated by summing the individual item scores included in the instrument after the factor analysis. The summative scores of the instrument were expressed as a mean and standard deviation (SD) and were compared using Student’s t‑test (gender comparison) and analysis of variance (ANOVA) (year of study). The PSAI subscale scores were calculated by summing the item scores in each subscale. The latter were expressed as means and SDs and were compared for gender and year of study using ANOVA. Post-hoc pairwise comparisons using the Scheffé method were conducted on the ANOVAs with statistically significant mean differences, to explore these differences between pairs of groups. Effect sizes (Cohen’s d or η2 (for ANOVA comparisons)) are provided to give an indication of the magnitude of the difference between the groups for all comparisons and f‑values to find out if the means between two populations are significantly different.

Regression analysis was run to model the relationship between the overall PSAI and MSATU scores by fitting a linear equation to the observed data in order to measure to what extent there is a linear relationship between them. The relevant assumptions of normality, linearity, homoscedasticity, and absence of multicollinearity were tested to ensure valid results would be obtained.

## Results

### Participants

A total of 168 (91%) fourth-year students participated in the pilot study (2012) and completed the 35-item survey. Of the 619 students invited to participate in the main study (2013), 484 (78%) responded. Non-respondents (those who chose to not participate in the survey) did not differ from respondents with regard to gender or year of study (both *p*-values >0.05), based on data of class size and gender proportions. Factor analysis was performed using the 35-item survey completed by 652 students—186 students (2012) and 484 students (2013). Tab. 1 shows the demographic data for the 484 students who participated in the main study (2013) and who completed the 35-item survey and the 10 items included from the MSATU scale. Sixty-percent of respondents were female which aligns with the average gender representation per study year which varies from 54 to 65% as well as the national matriculation female-to-male ratios of approximately 55:45 [41, 42].

**Table 1:** Overall Perceptions of Social Accountability Instrument (PSAI) scores for 484 medical students by student characteristics

Student characteristic	No. (%) of students	Overall PSAI score, mean (SD) ^a,c^	*p*-value	f‑statistic	d.f.	Cohen’s d
*Gender (t-tests)*						
			<0.001	1.434	2	0.352
Male	192 (40)	105.86 (14.1)				
Female	289 (60)	110.74 (13.6)				
*Medical school year (ANOVA)*						
			0.246	1.409	2	0.139^b^
First	175 (36)	107.4 (15.76)				
Third	195 (40)	109.7 (12.8)				
Sixth	114 (24)	109.25 (12.7)				
*Post hoc analysis*: no significant differences were found between groups on the overall PSAI scores						
*PSAI-MSATU scale scores (linear regression)*						
ANOVA			<0.001	148		
VIF		1.000				
R^2^		0.135				
Durbin-Watson		1.973				

^a^ Data are for the 484 first year, third, and sixth year students at the University of Cape Town Medical School who responded to the complete survey in 2013. Not all of these students provided demographics and other types of data, so the total number does not add up to 484 for each characteristic

^b^ η2

^c^ Possible overall PSAI scores range from 28–140

### Exploratory factor analysis

Tests of factorability suggested that the survey data were appropriate for EFA; the Kaiser-Meyer-Olkin value of 0.92 (above 0.5) and the Bartlett test of sphericity were statistically significant (χ^2^ = 7101.723, d.f. = 595, *p* < 0.0001). In addition, a respondent: item ratio of 18:1 (652 respondents and 35 items) was considered adequate for factor analysis (>10:1) [43]. Six items did not load on any factor at the 0.3 loading threshold and one item was excluded due to a low individual measure of sampling adequacy (MSA) in addition to very low correlations with other items. After removal of this item, the remaining 29 items represented an eight-factor solution with a cumulative variance of 54%. The scree plot and Kaiser criteria suggested that six factors should be retained. The Eigenvalues (and percentage variance) for these factors were 9.371 (28%), 1.741 (5%), 1.593 (5%), 1.426 (4%), 1.177 (4%), and 1.146 (3%), respectively. One item loaded on a factor by itself, leaving a total of 28 items on 5 factors which comprised the final factor structure hereafter referred to as the Perceptions of Social Accountability Instrument (PSAI) as is shown in Table 1 of the electronic supplementary material, and is consistent with the conceptual aspects of a multidimensionality notion of social accountability.

Review of the items included in the respective subscales showed that they reflected the main themes of advocacy and community; collaboration and engagement; leadership and professionalism; communication and patient-centred care; and medical expert and scholar. All of the 7 items which did not load or which were removed had originally belonged to the medical expert and collaborator role with the exception of one which was originally part of the manager role. Table 2 of the electronic supplementary material shows the correlations between the factor pairs and the Cronbach alpha for each factor/subscale.

### PSAI overall scores

**Fig. 1:**
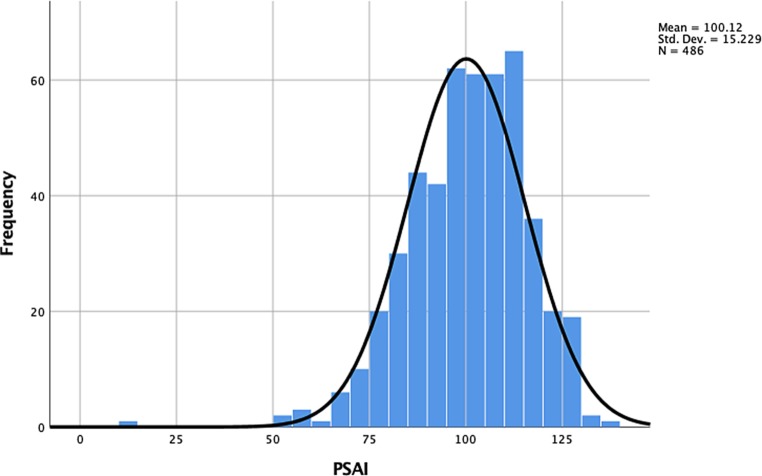
PSAI score for respondents, % (*n* = 484; possible overall PSAI scores range from 28–140)

Each of the retained 28 items was rated using a 5-point scale. The overall instrument score range was from 28 to 140. The higher scores indicated a greater appreciation of the importance of the competencies included in the instrument. The histogram in Fig. 1 shows that the overall scores were right shifted with a range from 50 to 138 and a mean score of 100.1 (SD = 15.3). Tab. 1 shows the overall scores disaggregated by gender and year of study. There was a significant difference between men and women (*p* = 0.000) but no significant difference was found between students in different years of study.

### PSAI subscale scores

Of the subscale scores, the communication and patient-centred care, expert and scholar, and advocacy dimensions had the highest average mean scores (reported as percentages of the total possible score of each subscale for ease of comparison), and engagement and collaboration the lowest (Table 3 of the electronic supplementary material). The mean subscale scores disaggregated by gender, and year of study are shown in Table 3 of the electronic supplementary material. Significant differences were observed between men and women for all subscales with the exception of expert and scholar, at the *p* < 0.5 level. Significant differences (*p* < 0.5) were observed between years of study for the subscales of communication and patient-centred care (particularly between respondents in year 1 and 3, and years 1 and 6).

### Relationship between PSAI overall scores and MSATU scores

An initial scatterplot showed a linear relationship between the two variables with no outliers. To show independence of observations, the Durbin-Watson statistic was checked. The normal predicted probability plot was used to confirm that the residuals were normally distributed (Fig. 1 of the electronic supplementary material), and also equally distributed (homoscedasticity). The variance inflation factor (VIF) value was not needed as there was only one predictor variable. A simple linear regression was calculated to predict the PSAI scores based on the MSATU scores. A significant regression equation was found indicating that the regression model statistically significantly predicts the outcome variable (i.e., it is a good fit for the data) even though the R^2^ value is considered low. The results of the regression analysis are presented in Table 1 and Fig. 1 of the electronic supplementary material.

## Discussion

This study describes the design of an instrument to evaluate medical students’ perceptions of the essential competencies of a socially accountable healthcare practitioner, as broadly articulated in the CanMEDS framework. The purpose was to develop a tool which could be used in the future to longitudinally evaluate the dynamic impact of curriculum design on trainees’ perceptions of the desired competencies of a socially accountable healthcare practitioner.

The study yielded a 28-item instrument, called the Perceptions of Social Accountability Instrument (PSAI), with five factors suggesting a multidimensional concept. The resulting dimensions correlate to all of the seven roles originally described in CanMEDS, i.e. medical expert, advocate, communicator, collaborator, scholar, professional, and manager/leader, although two (namely medical expert and collaborator) were mostly deconstructed, collapsed and subsumed into others. Our students, therefore, endorse the key competencies of a socially accountable healthcare practitioner as defined by a large body of healthcare professionals. This finding broadly agrees with previous studies exploring students and health professionals’ perceptions of the importance of the CanMEDS competencies [44].

In this study, the dimensions of communication and patient-centred care, expert and scholar, and advocacy were most highly rated by respondents. These findings both contrast and are supported by other studies where the advocacy role received the lowest rating and communicator and collaborator were more highly rated [25, 45]; the latter had the lowest scoring in this study. In addition, there were significant gender differences: women rated most of the subscales more highly than men. The observed influence of gender on the rating of advocacy is consistent with the literature and data from other scales which could be considered markers of advocacy [46]. Communication was also better appreciated by women students in our study. This finding is similar to previous studies showing that women physicians have more positive and appreciative attitudes towards communication and communication skills and demonstrate greater patient care [47, 48]. Similarly the gender differences in leadership and professionalism are also supported by other studies which show that women value professionalism and learning about ethics more than men [49]. A key challenge with measuring attitudes towards, and perceptions of professionalism, however, is the lack of widespread agreement on a definition despite the vast literature on the topic [50].

This study found significant differences between respondents in different years of study on the subscale communication and patient-centred care. Other published work has shown a progressive decline in medical student empathy and caring for the underserved during undergraduate training [15, 51, 52]. In contrast, this study found that the students in years 3 and 6 valued patient-centred care and communication more highly than those in the first year. This may be due to the limited interaction which first-year students have with patients and others in the healthcare setting leading to less appreciation of this competency. This finding is particularly surprising in light of the vast literature showing declines in empathy—of which patient-cared care and interaction are markers—as students progress through their studies, although as Mahoney et al.[53] report, the evidence is mixed and other studies have found that senior students have higher levels of empathy than junior counterparts [54]. Another South African study conducted with third-year medical students using the Jefferson Scale of Empathy instrument found significant differences in scores for students aged 25+ compared with younger students [55] suggesting that age may be a contributing factor to why third- and final-year students rated this higher than their first-year counterparts in this study.

Aggregate scores on the engagement and collaboration dimension were the lowest indicating that students did not value this as greatly as they did the other subscales. This may be partly explained, as discussed by Katoue et al. [56]: ‘*as part of their professional culture, physicians are usually trained to be self-sufficient and individually responsible for their decisions and actions’* perhaps resulting in students not appreciating the value of teamwork and engagement with stakeholders. Although the difference was not significant, it may explain why senior students’ scores were higher than those of the first-year students—they have had more exposure to the clinical setting and role models. The differences in scores between males and females are supported by other studies which show a more positive attitude among female students towards collaboration, cooperation and teamwork [57, 58].

South African medical students gain extensive experience caring for underserved populations who have limited financial means, i.e. 27% are unemployed, 22% have an annual household income of less than 300 US Dollars and 85% do not have any form of health insurance [59]. This experience working with this segment of the population may influence their perspectives regarding healthcare for indigent people, as compared with students who train in wealthier nations where unemployment rates are much lower and some form of medical insurance is the norm [60]. Further studies using our instrument in a range of variably resourced countries may uncover systemic differences in perspectives towards caring for underserved communities.

Use of the MSATU scale, which gives some indication of students’ social responsiveness, provides limited evidence of the construct validity of the instrument proposed in this paper. We only studied one external measure because the study was designed to develop an instrument which, once published, could be subjected to external validation in socially diverse settings. Social justice scales [61] may be useful in this regard but none have been assessed using diverse or large samples, an already recognised limitation [62].

### Limitations

This was a single-centre study, and while the instrument is based on the widely endorsed CanMEDS framework, it needs to be evaluated in variably resourced settings which may yield different results. Our study provides a cross-sectional ‘snapshot’ of students’ opinions, and further work is needed to obtain a longitudinal view as students progress through medical school. The assumption of normality of data was not met in all analyses in this study. However, the use of parametric tests for comparison of means is recommended when study sample sizes are large, i.e. ‘robust’ datasets [63]. Further validity evidence should also be sought on test content through expert review of the items and testing the instrument across relevant samples and contexts.

## Conclusions

This study describes the design of an instrument to evaluate medical students’ perceptions of the essential competencies of a socially accountable doctor, an understanding of which may enhance health sciences curriculum development. The results show that students value the domains of communication and patient-centred care, as well as advocating for patients and communities suggesting that the curriculum and training experiences may be successfully engaging students on these relevant issues and inculcating the desired values of future healthcare professionals. While the instrument does not purport to directly ‘measure’ social accountability, it may provide a longitudinal view of the impact of our training strategies on students’ perceptions of the essential competencies of socially accountable healthcare practitioners. Such data may provide evidence of the successes of our programs and identify areas where further improvements are required. The curriculum remains the most important vehicle for the achievement of socially accountable students, and their feedback is crucial to its development and medical schools’ success in achieving its mandate of preparing health professionals for the needs of the future.

## Caption Electronic Supplementary Material

Table 1. The 28 items and six subscales of the Perceptions of Social Accountability Inventory (PSAI): Exploratory Factor Analysis of 634 Medical Students’ Scores

Table 2. Observed correlations between Perceptions of Social Accountablity Instrument (PSAI) factors with cronbach alpha coefficient for each factor in parentheses

Table 3. Perceptions of Social Accountability Instrument (PSAI) Subscale Scores by Medical Student Characteristics

Fig. 1. The normal Predicted Probability plot between PSAIS overall scores and MSATU scores

## References

[1] Frenk J, Chen L, Bhutta ZA (2010). Health professionals for a new century: transforming education to strengthen health systems in an independent world. Lancet.

[2] Boelen C, Heck J. Defining and measuring social accountability of medical schools. 2018. http://whqlibdoc.who.int/hq/1995/WHO_HRH_95.7.pdf. Accessed October 24 2018.

[3] The Training for Health Equity Network. THEnet’s Social accountability evaluation framework Version 1. Monograph I (1 ed). The Training for Health Equity Network, 2011.

[4] Meili R, Ganem-Cuenca A, Leung JW, Zaleschuk D (2011). The CARE Model of Social Accountability: Promoting Cultural change. Acad Med.

[5] World Health Organization (2013). Transforming and scaling up health professionals’ education and training.

[6] Sandhu G, Garcha I, Sleeth J, Yeates K, Walker GRAIDER (2013). A model for social accountability in medical education and practice. Med Teach.

[7] ASPIRE. Aspire recognition of excellence in social accountability of a medical school: An Introduction. 2018. http://www.aspire-to-excellence.org/Areas+of+Excellence/. Accessed October 24 2018.

[8] WHO. Global strategy on human resources for health: workforce 2030. 2019. http://apps.who.int/iris/bitstream/10665/250368/1/9789241511131-eng.pdf. Accessed January 4 2019.

[9] Woollard RF (2006). Caring for a common future: medical schools’ social accountability. Med Educ.

[10] Leinster S (2011). Evaluation and assessment of social accountability in medical schools. Med Teach.

[11] Mcrea ML, Murdoch-Eaton D (2014). How do undergraduate medical students perceive social accountability?. Med Teach.

[12] Boelen C. The five-star doctor: An asset to health care reform? Geneva: World Health Organization. 2018. http://www.who.int/hrh/en/HRDJ_1_1_02.pdf. Accessed June 23 2018.

[13] Murdoch-Eaton D, Whittle S (2012). Generic skills in medical education: developing the tools for successful lifelong learning. Med Educ.

[14] Maudsley G, Williams EMI, Taylor DCM (2007). Junior medical students’ notions of a ‘good doctor’ and related expectations: a mixed methods study. Med Educ.

[15] Crandall SJS, Volk RJ, Cacy DA (1997). Longitudinal investigation of medical student attitudes toward the medically indigent. Teach Learn Med.

[16] Dunham L, Dekhtyar M, Gruener G (2017). Medical student perceptions of the learning environment in medical school change as students transition to clinical training in undergraduate medical school. Teach Learn Med.

[17] Zinn WM, Sullivan AM, Zotov N (2001). The effect of medical education on primary care orientation: results of two national surveys of students’ and residents’ perspectives. Acad Med.

[18] Philips JP, Wilbanks DM, Rodriguez-Salinas DF, Doberneck DM (2019). Speciality income and career decision-making: a qualitative study of medical student perceptions. Med Educ.

[19] Boelen C, Woollard B (2011). Social accountability: The extra leap to excellence for educational institutions. Med Teach.

[20] Parlow J, Rothman AIATSIM (1974). a scale to measure attitudes toward psychosocial factors in health care. Acad Med.

[21] Crandall SJ, Volk RJ, Loemker V (1993). Medical students’ attitudes toward providing care for the underserved: are we training socially responsible physicians?. JAMA.

[22] Harris P, Snell L, Talbot M, Harden RM (2010). for The International CBME Collaborators. Competency-based medical education: implications for undergraduate programs. Med Teach.

[23] Frank JR, Danoff D (2007). The CanMEDS initiative: implementing an outcomes-based framework of physician competencies. Med Teach.

[24] Van Heerden B (2013). Effectively addressing the health needs of South Africa’s population: The role of health professions education in the 21st century. S Afr Med J.

[25] Rademakers J, De Rooy N, Cate OT (2007). Senior medical students’ appraisal of CanMEDs competencies. Med Educ.

[26] Dharamsi S, Ho A, Spadafora SM, Woollard R (2011). The physician as health advocate: Translating the quest for social responsibility into medical education and practice. Acad Med.

[27] Buchman S, Woollard R, Meili R, Goel R (2016). Practising social accountability: From theory to action. Can Fam Phys.

[28] Wong C-S, Law KS, Huang GE (2008). On the importance of conducting construct-level analysis for multidimensional constructs in theory development and testing. J Manag.

[29] Ryan K, Gannon-Slater N, Culbertson MJ (2012). Improving survey methods with cognitive interviews in small- and medium-scale evaluation. Am J Eval.

[30] Coste J, Guillemin F, Pouchot J, Fermanian J (1997). Methodological approaches to shortening composite measurement scales. J Clin Epidemiol.

[31] Robinson GF, Switzer GE, Cohen ED (2013). A shortened version of the Clinical Research Appraisal Inventory: CRAI-12. Acad Med.

[32] Eller LS, Lev EL, Bakken LL (2014). Development and testing of the Clinical Research Appraisal Inventory-Short Form. J Nurs Meas.

[33] Joppe M. The Research Process. 2006. http://www.ryerson.ca/~mjoppe/rp.htm. Accessed September 28 2018.

[34] Validity DS (2003). on the meaningful interpretation of assessment data. Med Educ.

[35] Steiner DL, Validity NGR (2008). In Health Measurement Scales: A practical guide to their development and use.

[36] Watson JC (2007). Establishing evidence for internal structure using exploratory factor analysis. Meas Eval Couns Dev.

[37] Krabbe PFM, Validity I, Krabbe PFM (2017). The measurement of health and health status.

[38] Russell DW (2002). In search of underlying dimensions: The use (and abuse) of factor analysis in PSPB. Person Soc. Psychol Bull.

[39] Law KS, Wong CS, Mobley WH (1998). Toward a taxonomy of multidimensional constructs. Acad Manag Rev.

[40] Edwards J (2001). Multidimensional constructs in organizational behaviour research: An integrative analytical framework. Organ Res Method.

[41] Department of Education (2011). Education statistics in South Africa.

[42] Times Live. Matric girls fare better than boys. 2018. https://www.timeslive.co.za/news/south-africa/2018-01-04-matric-girls-fare-better-than-boys/. Accessed September 26 2019.

[43] Williams B, Onsman A, Brown T (2010). Exploratory factor analysis: A five-step guide for novices. J Emerg Prim Health Care.

[44] Norman G (2011). CanMEDS and other outcomes. Adv Health Sci Educ.

[45] Ortwein H, Knigge M, Rehberg B, Hein OV, Spies C (2011). Validation of core competencies during residency training in anaesthesiology. Ger Med Sci.

[46] Woloshuk W, Harasym PH, Temple W (2004). Attitude change during medical school: a cohort study. Med Educ.

[47] Wright KB, Bylund C, Ware J, Parker P, Query JL, Baile W (2006). Medical student attitudes toward communication skills training and knowledge of appropriate provider-patient communication: A comparison of first-year and fourth-year medical students. Med Educ.

[48] Löffler-Stastka H, Seitz T, Billeth S, Pastner B, Preusche I, Seidman C (2016). Significance of gender in the attitude towards doctor-patient communication in medical students and physicians. Wien Klin Wochenschr.

[49] Johnston J, Cupples M, McGlade K, Steele K (2011). Medical students’ attitudes to professionalism: an opportunity for the GP tutor?. Educ Prim Care.

[50] Birden H, Glass N, Wilson I (2014). Defining professionalism in medical education: A systematic review. Med Teach.

[51] Crandall SJ, Reboussin BA, Michielutte R, Anthony JE, Naughton MJ (2007). Medical students’ attitudes toward underserved patients: a longitudinal comparison of problem-based and traditional medical curricula. Adv Health Sci Educ.

[52] Neumann M, Scheffer C, Tauschel D, Lutz G, Wirtz M, Edelhäuser F (2012). Physician empathy: Definition, outcome-relevance and its measurement in patient care and medical education. GMS Z Med Ausbild.

[53] Mahoney S, Sladek R, Neild T (2016). A longitudinal study of empathy in pre-clinical and clinical medical students and clinical supervisors. BMC Med Educ.

[54] Magalhães E, Salgueira AP, Costa P, Costa MJ (2011). Empathy in senior year and first year medical students: A cross-sectional study. BMC Med Educ.

[55] Archer E, Turner R (2019). Measuring empathy in a. Group South African Undergrad Med Students Using Student Version Jefferson Scale Empathy Afr J Prm Health Care Fam Med.

[56] Katoue MG, Awad AI, Al-Jarallah A, Al-Ozairi E, Schwinghammer TL (2017). Medical and pharmacy students’ attitudes towards physician-pharmacist collaboration in Kuwait. Pharm Prac.

[57] Hansson A, Arvemo T, Marklund B, Gedda B, Mattsson B (2010). Working together—primary care doctors’ and nurses’ attitudes to collaboration. Scand J Public Health.

[58] Wilhemsson M, Ponzer S, Dahlgren L‑O, Timpka T, Faresjö. Are female students in general and nursing students more ready for teamwork and interprofessional collaboration in healthcare? BMC Med Educ. 2011;11:15. 10.1186/1472-6920-11-15 PMC3110123 21510872

[59] StatSA. Employment, unemployment, skills and economic growth. 2018. http://www.statssa.gov.za/presentation/Stats%20SA%20presentation%20on%20skills%20and%20unemployment_16%20September.pdf. Accessed October 31 2018.

[60] Health Systems Trust. South African Health Review. 2018. https://www.health-e.org.za/wp-content/uploads/2015/10/HST-SAHR-2014-15-Complete.pdf. Accessed October 31 2018.

[61] Torres-Harding SR, Siers B, Olson BD (2012). Development and psychometric evaluation of the Social Justice Scale (SJS). Am J Commun Psychol.

[62] Fietzer AW, Ponterotto J (2015). A Psychometric Review of Instruments for Social Justice and Advocacy Attitudes. J Soc Action Couns Psychol.

[63] Norman G (2010). Likert scales, levels of measurement and the “laws” of statistics. Adv Health Sci Edu.

