# Supplementary material for: Development of a CanMEDS-based instrument for evaluating medical students’ perceptions of the key competencies of a socially accountable healthcare practitioner
Source: Perspect Med Educ. 2020 Feb 7;9(2):98–106. doi: 10.1007/s40037-020-00564-6 (PMC7138770; doi:10.1007/s40037-020-00564-6)
Supplement: Supplementary file 1 — Table 1. The 28 items and six subscales of the Perceptions of Social Accountability Inventory (PSAI): Exploratory Factor Analysis of 634 Medical Students’ Scores [file 40037_2020_564_MOESM1_ESM.docx]

| **Table 1. The 28 items and six subscales of the Perceptions of Social Accountability Inventory (PSAI): Exploratory Factor Analysis of 634 Medical Students’ Scores** | | | | | | | | |
| --- | --- | --- | --- | --- | --- | --- | --- | --- |
|  |  |  | Factor loadings | | | | | |
| Subscale | Attribute | Item | 1 | 2 | 3 | 4 | 5 | 6 |
| Advocacy | M3 | Participate in activities aimed at controlling healthcare costs | .514 |  |  |  |  |  |
|  | A3 | Participate in activities that aim to improve living conditions for the poor | .824 |  |  |  |  |  |
|  | ME4 | Take responsibility for disease prevention in the community | .495 |  |  |  |  |  |
|  | A4 | Participate in activities that promote free healthcare for the poor | .792 |  |  |  |  |  |
|  | S4 | Participate in patient-education activities | .551 |  |  |  |  |  |
|  | A5 | Identify and respond to marginalized communities | .629 |  |  |  |  |  |
|  | A1 | Participate in social justice activities | .479 |  |  |  |  |  |
|  | CM4 | Dedicate time to health promotion activities | .504 |  |  |  |  |  |
|  | A2 | Identify the determinants of health of the community served | .460 |  |  |  |  |  |
| Communication & Patient-centred | CM1 | Effective communication with patients, colleagues etc. |  | .418 |  |  |  |  |
|  | M1 | Ensure that treatment is cost effective |  | .335 |  |  |  |  |
|  | CM3 | Provide information to patients to empower them to make decisions |  | .579 |  |  |  |  |
|  | CM2 | Allow patients to participate in decision making about their care |  | .511 |  |  |  |  |
|  | ME2 | Understand the influence of social/cultural factors on illness |  | .418 |  |  |  |  |
| Leader & Professional | P2 | Seek help when dealing with personal/professional difficulties |  |  | .348 |  |  |  |
|  | P4 | Consult colleagues when faced with ethical dilemmas |  |  | .305 |  |  |  |
|  | M2 | Serve as leaders in healthcare provision |  |  | .530 |  |  |  |
|  | P1 | Set a positive example regarding social responsibilities |  |  | .534 |  |  |  |
|  | CL3 | Consult other team members when changing treatment plans |  |  | .355 |  |  |  |
| Engagement & Collaboration | M5 | Develop organisational management skills |  |  |  |  | .506 |  |
|  | S5 | Mentor and teach colleagues |  |  |  |  | .506 |  |
|  | P5 | Devote time to recreational/professional activities other than medical career |  |  |  |  | .445 |  |
|  | CM5 | Present medical information to the public and media |  |  |  |  | .598 |  |
| Expert & Scholar | S1 | Engage in lifelong learning |  |  |  |  |  | .434 |
|  | S2 | Apply evidence-based medicine in clinical practice |  |  |  |  |  | .378 |
|  | S3 | Develop research skills to advance medical knowledge |  |  |  |  |  | .367 |
|  | ME1 | Knowledge of clinical science |  |  |  |  |  | .328 |
|  | P3 | Spend time thinking about the quality of healthcare you provide |  |  |  |  |  | .316 |
| Factor analysis used the responses of 634 first, third, fourth and sixth year medical students.  Factor loadings<0.30 are suppressed (ME5, CL5,ME3, CL2).  Likert scale for responses (unless otherwise designated); strongly agree, agree, neutral, disagree, strongly disagree.  Items CL1 & M4 removed and excluded from analysis.  CL4 loaded by itself on a single factor | | | | | | | | |
