# Supplementary material for: Development of a CanMEDS-based instrument for evaluating medical students’ perceptions of the key competencies of a socially accountable healthcare practitioner
Source: Perspect Med Educ. 2020 Feb 7;9(2):98–106. doi: 10.1007/s40037-020-00564-6 (PMC7138770; doi:10.1007/s40037-020-00564-6)
Supplement: Supplementary file 2 — Table 2. Observed correlations between Perceptions of Social Accountablity Instrument (PSAI) factors with cronbach alpha coefficient for each factor in parentheses [file 40037_2020_564_MOESM2_ESM.docx]

| **Table 3: Observed Correlations Between Perceptions of Social Accountability Instrument (PSAI)**  **Factors With Cronbach Alpha Coefficient for Each Factor in Parentheses** | | | | | |
| --- | --- | --- | --- | --- | --- |
| **Factor** | **1** | **2** | **3** | **4** | **5** |
| 1 | (88) | .395 | .492 | .509 | .494 |
| 2 |  | (50) | .427 | .351 | .439 |
| 3 |  |  | (66) | .453 | .477 |
| 4 |  |  |  | (70) | .518 |
| 5 |  |  |  |  | (56) |
