# Supplementary material for: Development of a CanMEDS-based instrument for evaluating medical students’ perceptions of the key competencies of a socially accountable healthcare practitioner
Source: Perspect Med Educ. 2020 Feb 7;9(2):98–106. doi: 10.1007/s40037-020-00564-6 (PMC7138770; doi:10.1007/s40037-020-00564-6)
Supplement: Supplementary file 3 — Table 3. Perceptions of Social Accountability Instrument (PSAI) Subscale Scores by Medical Student Characteristics [file 40037_2020_564_MOESM3_ESM.docx]

| **Table 3: Perceptions of Social Accountability Instrument (PSAI) Subscale Scores by Medical Student Characteristics** | | | | | | | | |
| --- | --- | --- | --- | --- | --- | --- | --- | --- |
|  | **PSAI Subscale score, mean (SD)*** | | | | | | | |
| **Student Characteristic** | **Advocacy** | **Engagement &**  **Collaboration** | **Leader &**  **Professional** | **Communication & Patient-centred** | | | **Expert & Scholar** |  |
| **Overall subscale scores**** | **79.03** | **85.89** | **76.91** | **75.35** | | | **85.55** |  |
| **Gender (t-tests)** | | | | | | | | |
| Male (n=192) | 30.3 (6.6) | 14.7 (2.9) | 18.8 (3.3) | 20.9 (2.8) | | | 12.4 (4.9) |  |
| Female (n=290) | 32.48 (6.0) | 15.3 (2.9) | 19.5 (3.2) | 21.9 (2.6) | | | 13.2 (4.8) |  |
| *p*-value | **<0.001** | **0.026** | **0.017** | **<0.001** | | | 0.057 |  |
| f-statistic | 2.447 | .0299 | .194 | 1.594 | | | 0.00 |  |
| D.o.F | 1 | 1 | 1 | 1 | | | 1 |  |
| *Cohen*’s d | 0.345 | 0.206 | 0.215 | 0.37 | | | 0.165 |  |
| Mean value (percentage mean value) | 31.61 (79.03) | 15.07 (75.35) | 19.23 (76.92) | 21.47 (85.9) | | | 21.39 (85.55) |  |
|  | | | | | | | | |
| **Medical school year (anova)** | | | | | | | | |
| First | 30.79 (7.03) | 14.82 (3.2) | 19.35 (3.07) | 20.93 (3.07) | | | 10.66 (2.74) |  |
| Third | 32.3 (5.89) | 15.22 (2.8) | 19.13 (3.05) | 21.72 (2.47) | | | 16.26 (5.7) |  |
| Sixth | 31.71 (5.85) | 15.2 (2.63) | 19.21 (3.01) | 21.86 (2.49) | | | 10.79 (2.23) |  |
| *p*-value | 0.72 | 0.37 | 0.81 | **0.04** | | | 0.73 |  |
| f-statistic | 2.647 | 0.989 | 0.211 | 5.625 | | | 0.320 |  |
| D.o.F | 2 | 2 | 2 | 2 | | | 2 |  |
| Effect size ( η2) | 0.105 | 0.06 | 0.04 | 0.04 | | | 0.03 |  |
| Post-hoc analysis*** |  |  |  | 1 | 3  6 | 0.021  0.014 |  |  |
|  |  |  |  | 3 | 1  6 | 0.021  0.871 |  |  |
|  |  |  |  | 6 | 1  3 | 0.014  0.871 |  |  |
| * Data are for the 484 first year, third, and sixth year students at the University of Cape Town Medical School who responded to the complete survey in 2013. Not all of these students provided demographics and other types of data, so the total number does not add up to 484 for each characteristic.  ** Percentage for each subscale calculated based on mean value expressed as percentage of total possible score for the subscale  *** Scheffe method, only significant differences shown. | | | | | | | | |
