# Supplementary figures and images for: Development of a CanMEDS-based instrument for evaluating medical students’ perceptions of the key competencies of a socially accountable healthcare practitioner
Source: Perspect Med Educ. 2020 Feb 7;9(2):98–106. doi: 10.1007/s40037-020-00564-6 (PMC7138770; doi:10.1007/s40037-020-00564-6)

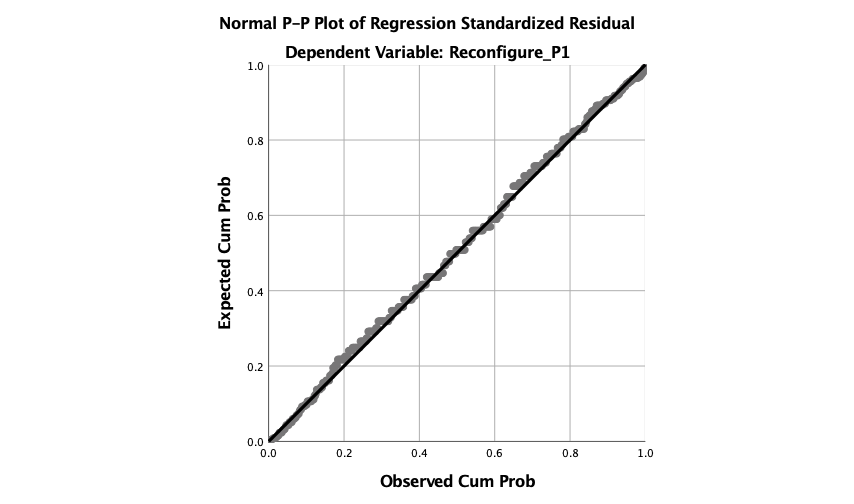


Fig. 1.

Supplement: Supplementary file 4 — Fig. 1. The normal Predicted Probability plot between PSAIS overall scores and MSATU scores [file 40037_2020_564_MOESM4_ESM.docx]
